# Tissue Engineering of the Urethra: From Bench to Bedside

**DOI:** 10.3390/biomedicines9121917

**Published:** 2021-12-15

**Authors:** Dusan Pastorek, Martina Culenova, Maria Csobonyeiova, Veronika Skuciova, Lubos Danisovic, Stanislav Ziaran

**Affiliations:** 1 Department of Urology, University Hospital Bratislava, Pazitkova 4, 821 01 Bratislava, Slovakia; dusan.pastorek@fmed.uniba.sk; 2 Institute of Medical Biology, Genetics and Clinical Genetics, Faculty of Medicine, Comenius University, Sasinkova 4, 811 08 Bratislava, Slovakia; martina.culenova@fmed.uniba.sk (M.C.); lubos.danisovic@fmed.uniba.sk (L.D.); 3 National Institute of Rheumatic Diseases, Nabrezie I. Krasku 4, 921 12 Piestany, Slovakia; 4 Institute of Histology and Embryology, Faculty of Medicine, Comenius University, Sasinkova 4, 811 08 Bratislava, Slovakia; maria.csobonyeiova@fmed.uniba.sk; 5 Department of Histology and Embryology, Jessenius Faculty of Medicine in Martin, Comenius University, Mala Hora 4, 036 01 Martin, Slovakia; veronika.skuciova@unilabs.sk; 6 Department of Urology, Faculty of Medicine, Comenius University, Limbova 5, 833 05 Bratislava, Slovakia

**Keywords:** tissue engineering, urethra, urethral stricture, urethral reconstruction, stem cells, scaffolds

## Abstract

Tissue engineering (TE) is a promising approach for repair/substitution of damaged tissues and organs. Urethral strictures are common and serious health conditions that impair quality of life and may lead to serious organ damage. The search for ideal materials for urethral repair has led to interest of scientists and surgeons in urethral TE. Over the last decades, a significant amount of preclinical studies and considerable progress have been observed. In contrast, urethral TE has made slow progress in clinical practice so far. To address this, we conducted a systematic review of the literature on clinical applications of TE constructs for urethral repair in the last three decades. In summary, the TE approach is promising and effective, but many issues remain that need to be addressed for broader adoption of TE in urethral repair. Better design of trials, better cooperation of research groups and centralization could lead to reduction of costs and slowly proceed to commercialization and routine use of TE products for urethral reconstruction.

## 1. Introduction

Tissue engineering (TE) is an interdisciplinary field, which combines elements from biology, material science, medicine and engineering to produce new approaches and therapies for tissue and organ regeneration. It refers to the use of building blocks comprised of cells and scaffolds, either derived from extracellular matrix (ECM) or synthetic materials for tissue repair [1]. Scaffolds are defined as materials that have been engineered to cause desirable cellular interaction. They serve as a support for cells but also provide a biochemical and physical environment similar to native tissue [2].

The urethra is the duct connecting the urinary bladder to the body exterior to produce urine. In males, it is a part of the genital tract as well. Due to the significant differences between male and female urethra, the male urethra consists of functionally and anatomically defined parts (prostatic, membranous, spongious urethra containing the bulbar urethra and the penile urethra) [3,4]. Urethra may be affected by many pathological processes and thus negatively affect the quality of life or even lead to organ impairment. For example, congenital birth defects of urethra, such as hypospadias (1 in every 300 births) [5], and acquired urethral abnormalities, such as urethral strictures (1 in every 1000 men > 65 year of age) are the most common [6]. Urethral strictures are most common among adults and most often occur as a result of scarring, which replaces the vascular tissue of the corpus spongiosum, leading to ischemic spongiofibrosis. Replacement of damaged urethra by scar tissue leads to a reduction of its lumen, with the gradual formation of lower urinary tract obstruction [7].

Treatment of strictures usually involves a surgical procedure such as urethral dilation (UD) or direct vision internal urethrotomy (DVIU). No statistically significant difference in surgical outcome between DVIU and UD was described by Steenkamp et al. [8], but both procedures become less effective with increasing stricture length. Patency rates vary considerably between 8% and 77% after DVIU [9,10].

The most important predictive factor for stricture recurrence is length of stricture. Steenkamp et al. indicated that with each 1 cm increase in the stricture length, the risk of recurrence is increased by 1.22 (95% CI: 1.05–1.43) [8]. In addition, in the systematic review of a case series, a weighted average patency rate was 71.2% vs. 23.2% for strictures less or more than 1 cm, respectively (*p* < 0.0001) [11].

Because of these drawbacks, EAU guidelines recommend not using DVIU/UD as solitary treatment for long (>2 cm) segment strictures. On the other hand, better long-term success rates are associated with an open reconstructive treatment, urethroplasty. These procedures are usually multi-staged interventions, often with the use of buccal or skin autologous grafts or flaps [12].

A systematic review by Mangera et al. [13] showed an average patency rate of 90.5% with the use of all types of grafts for staged penile urethroplasties with an average follow-up of 22.2 months. However, buccal mucosa harvesting is painful and not complication-free (bleeding, postoperative infection, pain, swelling, salivary duct disorders, restricted mouth opening, scar formation, contracture, loss of sensation due to nerve injury, impairment of mouth opening, smiling, whistling, diet and speech) [14,15,16].

Progress and development in TE has the potential to overcome these limitations. Despite huge progress in pre-clinical settings, clinical application of TE products in urethral repair remains challenging. The aim of this article is to review the current literature with a focus on clinical applications of TE products for urethral repair in the last three decades, the efficacy and the translational status of urethral TE.

## 2. Methods and Study Selection

**Figure 1:**
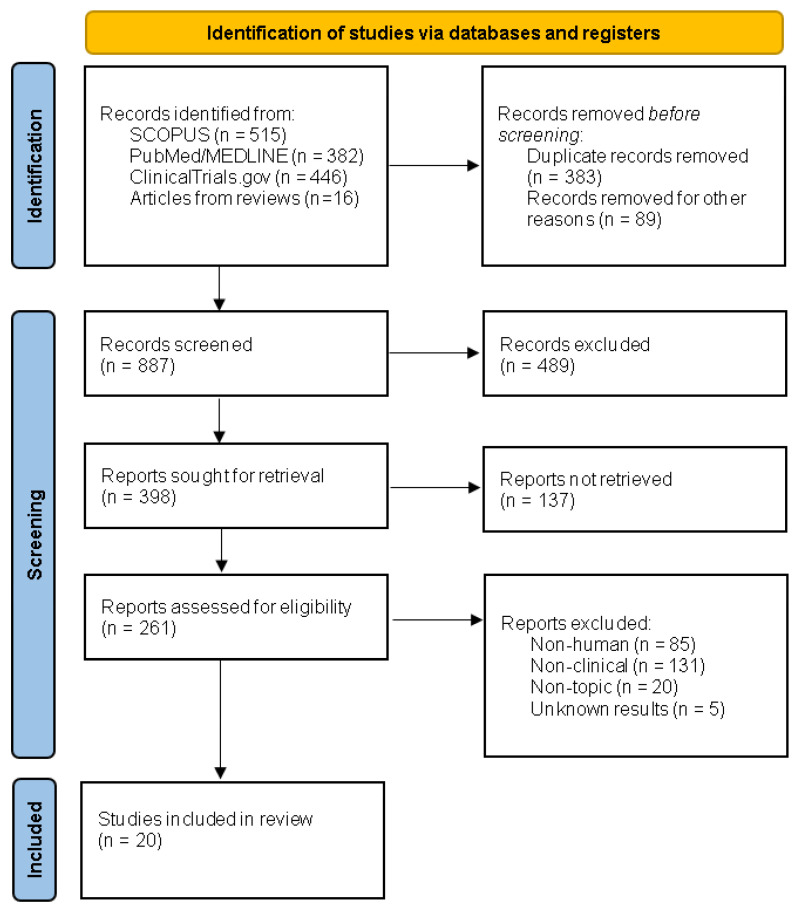
PRISMA flow diagram depicting the process of the literature search.

We followed the Preferred Reporting Items for Systematic Reviews and Meta-Analyses (PRISMA) guidelines. The study was registered in PROSPERO; registration number 296616. The search was performed (22 September 2021) in the databases of PubMed/Medline, Scopus and register ClinicalTrials.gov. Significant studies (*n* = 12) retrieved from the selected reviews were included. Study selection was restricted to the last 30 years, in humans and in English. Terms such as tissue engineering, urethra, urethral stricture, urethral reconstruction, stem cells and scaffolds were applied as the key words for the primary screening. Studies were assessed independently by SZ, DP and MC. At first, all retrieved results (*n* = 1359) were exported to MS office Excel 2019 and duplicates were removed (*n* = 383). Another 89 records were excluded as they did not represent original scientific articles. Subsequently, another sorting was performed based on the title, abstract, language and relevance. According to this, 261 articles were assessed for eligibility and exclusion criteria such as non-human, non-clinical or not topic-related. Finally, 35 articles were thoroughly screened and 22 were selected for review. Figure 1 illustrates the outline of the literature search in the form of a PRISMA flow diagram. As studies selected for the purpose of this review do not present consistent groups and vary in many parameters, further statistical analysis could not be performed.

## 3. Results

According to the aforementioned study selection process, 20 articles were selected to be included for this systematic review. Based on the chosen TE approach, we categorized obtained results into following groups: small intestinal submucosa grafts (*n* = 9), bladder-derived matrices (*n* = 3), acellular dermis graft (*n* = 1) and tissue TE approach (*n* = 7). Results are summarized in Table 1.

### 3.1. Small Intestinal Submucosa Grafts (SIS)

Altogether, SIS was applied in urethral reconstruction with the length varying from 0.5 up to 10 cm in nine studies. Studies vary greatly in terms of number of patients, site of strictures, follow up period and technique used (Table 1).

We found nine studies that used dorsal onlay technique for urethral repair. Patent urethral lumen with no evidence of stricture was used as a criterion for successful procedure in all studies. The first substitution urethroplasty using SIS was performed in 2003. However, this procedure was performed only on one patient with a history of long stricture of penile and bulbar urethra with the follow-up of 16 months. The patient had a satisfactory urodynamic–urine flow rate and the subjective outcome was reported as satisfactory [18]. A few years later, the same surgical technique was chosen in nine patients with 89% success rate [22]. One patient had stricture recurrence due to urinary infection, six patients reported having post-micturition dribbling. Le Roux et al. [20] used tubularized unseeded SIS graft in nine patients, which was implanted after DIVU and endoscopic urethroplasty. Two patients with patent lumen without any interventions after 2 years follow-up were reported, while stricture recurrence was identified in six patients; one patient was lost to follow-up. Overall, these results were concluded as unsatisfactory. With similar results, SIS was applied by Hauser et al. [23] in five patients and onlay urethroplasty was performed. Postoperative extravasation was present in one case; one patient developed severe urethritis. Recurrent stricture was reported in four patients during 17.5 months of follow-up.

Significantly better results in 50 patients were reported by Fiala et al. (24). Porcine SIS collagen-based matrix was used for bulbar, bulbopenile and the distal penile urethral strictures. Ventral onlay urethroplasty was performed, with a follow-up of 24–36 months; success rate was reported in 80% (40 patients), with no evidence of stricture recurrence. These occurred in the first 6 months postoperatively

Three different surgical techniques using SIS were described by Palminteri et al. [25]. With 14 patients recruited, dorsal inlay graft urethroplasty was performe; 1 patient underwent ventral onlay urethroplasty and 5 patients dorsal onlay plus ventral onlay graft urethroplasty. Mean follow-up was 21 months, no postoperative complications were noted, and successful outcomes were reported in 17 cases (85%). The same author later conducted a study with 25 men with bulbar strictures; the graft was placed dorsally in 11 patients, ventrally in 6 and ventrally plus dorsally in 8, with a mean follow-up of 71 months, with success rate of 76% (19 patients) and failure rate of 24% (6 patients) [32].

Farahat et al. [29] placed a SIS graft endoscopically for the treatment of short, recurrent inflammatory bulbar strictures in 10 patients, with a mean follow-up of 15 months. Following DIVU, SIS patch was introduced into the urethra endoscopically. It is noteworthy that this technique was used for treatment short strictures (0.5–2cm). Authors reported two cases of stricture recurrence (20%), which were managed by regular monthly urethral dilatation.

Successful application of SIS was demonstrated in a consecutive series of 28 patients. SIS was applied in an onlay and inlay fashion for the correction of anterior urethral strictures which were 3.5–7 cm long [33]. With a mean follow-up of 24.8 months, the success rate was 93% (26 patients), two patients developed strictures at 5 and 6 months respectively. Authors concluded that the use of SIS in this setting is a safe and effective reconstructive material for selective use in urethral reconstructive surgery.

### 3.2. Bladder-Derived Matrices

Collagen-based bladder matrices (*n* = 2) and acellular bladder matrix (*n* = 1) were used for urethral reconstruction of hypospadias or urethral strictures.

The pilot study by Atala et al. reported nine patients with a history of failed hypospadias repair [17]. Collagen-based inert matrix was chosen for the urethral reconstruction, and complications developed only in one patient who received a 15-cm-long segment of neo-urethra. Subglandular fistula was detected and treated by standard surgical techniques. All patients underwent urethrocystoscopy and histological examination 1 year postoperatively. After 22 months of follow-up (mean), adequate caliber conduits, normal appearing urethral tissue and typical urethral stratified epithelium were confirmed.

In the following study, bladder submucosa collagen-based inert matrix as free graft substitute for urethral stricture repair was used for 28 patients. Ventral onlay technique was used in all cases and the mean follow-up was 37 months. A total of 24 patients (86%) were rated as success. A slight caliber decrease at the anastomotic sites on urethrography was reported in four patients. In one case, subcoronal fistula was developed and closed spontaneously 1 year after the procedure [19]. Finally, a randomized comparative study was performed in order to compare acellular bladder matrix (BAMG) and buccal mucosa in 30 patients with urethral strictures. Results showed that BAMG had a 53% success rate, compared to the application of buccal mucosa graft, which had a 100% success rate. Authors divided these two groups into subgroups of patients with healthy and unhealthy urethral bed. In the subgroup of patients with healthy urethral bed (not undergone prior intervention for urethral stricture) there was a success rate of BAMG 89%. In the subgroup of patients with unhealthy urethral bed there was a success rate of BAMG only 33%. In case of buccal mucosa application, the success rate in both subgroups was 100%. Authors concluded that the use of BAMG is a viable option for urethral repair in patients with a healthy urethral bed and no spongiofibrosis [28].

### 3.3. Acellular Dermis Graft

Acellular dermis (AlloDerm, LifeCell Technologies, Maharashtra, India) was used only in one patient with severe comorbidities. However, this material was combined with buccal mucosa as staged therapy. During a 6-month follow-up time interval, the patient had no evidence of residual infection. Neourethra was functional and the patient was able to void normally [21].

### 3.4. Tissue Engineering Approach

TE grafts were used in nine studies. All of them used both biocompatible materials (scaffold) seeded with autologous cells for urethral stricture correction.

Fossum et al. investigated the efficacy of TE approach in six children with a history of severe hypospadias [26]. Autologous in vitro cultured urothelial cells were placed on acellular dermis and transplanted into affected tissue. Two patients were reported as success (33%); there was one case of a developed stricture which was treated conservatively with good effect. Two other patients suffered from fistula that required uneventful surgical correction and one patient developed an obstruction that required internal urethrotomy as well. Cosmetic appearance was reported as good in all cases and authors concluded this technique was feasible for treatment of this selected group of patients.

Another study evaluated the use of TE autologous grafts of cultured oral keratinocytes and fibroblasts which were seeded on sterilized donor de-epidermised dermis for reconstruction of the stricture associated with genital lichen sclerosis [27]. This approach was used in five patients. Full-thickness grafts were used in two cases for one-stage and in three cases for two-stage anterior urethroplasty. One patient required complete excision of the grafted area because of the tissue fibrosis. The other three patients required urethrotomy or dilatation. Despite the developed complications, authors considered TE buccal mucosa as a potential graft to be applied in clinical medicine.

A study by Raya-Rivera et al. [30] aimed to assess the effectiveness of TE urethras using patients’ autologous urothelial and smooth muscle cells. This is one of a few studies where autologous cells were seeded on the luminal (urothelial cells) and outer surface (muscle cells) of a tubularized polyglycolic acid mesh scaffold to mimic histologic composition of urethra. These constructs were used as urethral replacement grafts for severe urethral defects 4 to 6 cm long (history of failed posterior urethral repair or complete posterior urethral disruption from pelvic trauma) in five patients. After three months, biopsies showed a normal architecture of TE urethras and no aberrant histological changes were reported. Moreover, these excellent results were reported after a median follow-up of 71 months.

Autologous urothelial cells were seeded on the acellular dermis in order to treat scrotal or perineal hypospadias and pronounced chordee [31]. The study involved six patients, urethroscopy and biopsy of the neourethras were performed at 3–4 and 6–8 years postoperatively. All patients could void without straining and urethroscopy showed a well-formed and wide penile urethra without sacculation or diverticula.

The same material was used in five patients who underwent urethroplasty [27]. Excision of entire graft due to scarring was necessary in one patient, partial excision in one patient, and there was stricture recurrence in three patients.

MukoCell^®^ was applied in two studies. One study described using standard techniques such as ventral onlay, dorsal onlay, dorsal inlay and combined with MukoCell^®^ in 38 patients. This study reported 32 patients (84.2%) as success and 6 patients (15.8%) as failure due to the need to undergo further urethral reconstruction [34]. The second study was a multicenter, prospective, monitored non-interventional observational trial [35] which included 99 patients with recurrent urethral strictures. To manufacture the graft, biopsy of oral mucosa was harvested and submucosa was separated and used to establish primary culture of the epithelial cells. Success rates ranged between 85.7% and 0% depending on high or low surgical experience.

Mandal et al. [36] evaluated the use of an acellular TE bovine pericardial patch in augmentation urethroplasty for long segment urethral strictures. Dorsal onlay technique was used in nine cases. Reported success rate was 88.9% (eight patients).

## 4. Discussion

TE was met with enthusiasm in the late 20th century and truly exploded later. For example, in 2010, over 4000 articles were available on PubMed when searching “tissue engineering” or “regenerative medicine,” as compared to less than 400 in the beginning of the 21st century [37].

The main promise that TE holds is that it may overcome the most important problems in reconstructive urology, i.e., lack of appropriate material for urethral reconstruction. Moreover, TE grafts can be manufactured without the need of additional surgery that in turn reduces potential serious side effects associated with harvesting e.g., oral mucosa [38]. Surprisingly, we did not find any information or patient questionaries to evaluate patient preference between noninvasive collection of stem cells for TE construction and buccal mucosa harvesting with subsequent urethroplasty. Collection of autologous stem cells e.g., from urine, is feasible and opens new possibilities for TE in reconstruction surgery [39].

Another important advantage is the virtually unlimited quantity of biomaterial that can be transformed into a functional graft of any size [40]. This would save operative time and perioperative morbidity associated with graft or flap harvest for tissue substitution. Despite these advantages, clinical application is still modest to say at best.

In contrast, there has been a considerable number of preclinical trials published that have demonstrated variable results [41,42].

While many preclinical studies have been performed, TE is still not used as an alternative treatment in routine clinical practice, except for a select patient group with a history of failed repairs [43,44,45].

Potential advantages of regenerative techniques are now overlooked by several reasons. In summary, these reasons are: (i) quality of clinical studies, (ii) cost and complexity of TE constructs, (iii) regulatory issues and legislation aspects.

Quality and experimental design of clinical studies is insufficient. Except for one study [28], no control groups were present, there was no randomization and trials lacked study protocols. Most studies included in this review showed a heterogeneous patient population, and approximately 70% of patients had one or more previous treatments, e.g., DIVU or failed urethroplasty, before using TE constructs. Using TE products in complex cases harboring higher complication rates places TE in a disadvantageous position to prove itself. In most studies, TE constructs were used for the treatment of more complex and lengthy strictures, which could be hardly managed by buccal mucosa grafting and urethroplasty. This could also influence results and direct “head-to-head” comparison to “standard urethroplasties”.

Studies in this review included reporting of outcome measures, follow-up time, side effects, surgical procedure and size of strictures. Surprisingly, urethrography was seldom used during follow up, and this could provide additional information in terms of success rates. To improve the level of evidence, and subsequently to facilitate the use of TE products into practice, more randomized controlled trials (RCTs) are warranted.

For common use of TE in clinical practice, large RCTs must prove superiority or at least non inferiority over conventional treatment. RCT allows valid inferences considering cause and effect of clinical interventions [46]. Without proper RCTs, no direct comparisons with current clinical practice (buccal urethroplasty) can be made. A recent systematic review of urethral TE showed that complex two-stage urethroplasty has complication-free rates and functionality of approximately 62%, 67%, and 36% [47], which is similar to the outcome of TE urethras. This suggests that urethral TE may be a valid alternative for further investigation [41].

One of the important drawbacks of TE for investigation and development is that the trial of a TE construct is not simply a test of a new medicinal product. The development of TE constructs includes trials of a complete process in vitro, in vivo or ex-vivo (construction, extensive testing for cytotoxicity, biodegradability, biomechanics, implantation, follow-up, regenerative effect of the TE product in the patient, analysis, and the final functional outcome) [48]. Moreover, standardization of a surgical intervention is difficult; new TE products may have unique properties for the surgeon who conducts an implantation of the TE product. The surgical technique may be refined and changed over time due to the surgical learning curve. Consequently, in multicenter studies, the differences in skills and experiences of the operating teams may introduce further variation, provided there is not a robust number of patients [49]. This can be clearly seen in a multicenter study [35], where the outcome of the implanted TE constructs clearly depended on surgical experience.

There are three remarkable studies that need to be commented on. Firstly, El Kassaby et al. [28] conducted the first randomized comparative study between buccal mucosal and acellular bladder matrix grafts in complex anterior urethral strictures. This study enrolled 30 patients with a follow-up of 25 months, and buccal mucosal grafts outperformed acellular bladder matrix grafts (100% vs. 66%, two patients lost in follow up period). Even though the success rate of 66% is lower than average when comparing with buccal urethroplasty, this study used randomization with a sufficient patient sample.

Raya Rivera [30] et al. enrolled five boys with complex strictures with a follow-up of 71 months (mean) and 100% success rate. It is interesting that the authors used TE tubularized polyglycolic acid:poly(lactide-co-glycolide acid) scaffolds which were seeded with muscle and epithelial cells. Although this was a small series, it provided further evidence that regenerative medicine can provide enduring repairs in patients with complex strictures.

We believe that seeding appropriate tubularized scaffolds with autologous cells might be the right direction for TE construct application.

Ram-Liebing et al. [35] conducted a multicenter, prospective trial with 99 patients and mean a follow-up of 24 months. The results clearly depended on the center where the TE constructs were applied (two low volume centers reporting success rates of 0 and 50%, the others reported significantly better results). However, this is a pilot multicenter study where they used synthetic commercially available products (Mukocell) for urethral repair.

These articles represent the most important reports in the clinical use of TE materials for urethral reconstruction and could help to pave the way for routine clinical application of TE constructs for urethral repair.

The major disadvantage of the TE approach is the high cost of production and lack of off-the-shelf availability products. These limitations can be (and must be) overcome by continuous scientific developments with industry involvement. As was mentioned before, high-quality phase 1 and 2 studies are needed with long-term follow up, which should be followed by careful commercialization. Hand in hand, it is necessary to create multidisciplinary high-volume centers with appropriate experience in TE that follow GMP protocols. Establishing dedicated working groups of clinical and biotechnology experts should be the cornerstone of the transition of TE products into clinical practice. The clinical and research teams must work closely to coordinate the timing of cell harvesting, cell-seeding, TE construct maturation (bioreactors) and eventual urethroplasty. These interdisciplinary teams will be able to conduct trials (preclinical and clinical) in an effective way, based on simultaneous, continuous cooperation. This process is time consuming and expensive, but the cost-effectiveness of TE will ultimately improve after broader adoption of this technique. It is necessary to involve industry and financing to facilitate large-scale production of scaffolds and biomaterials based on standardized protocols. Determining the cost-effectiveness of TE products may be very complex because it is not easy to determine what to include in the calculation of costs of treatment. TE construction contains biologically active molecules and/or cells and its behavior in the body is less predictable than that of a medical device with “stable” and predictable properties [50].

When considering cost-effectiveness in urology, it is important to illustrate involvement of industry and urologic surgeons into robotic surgery. Even though robotic surgery is clearly not cost effective with comparable surgical outcomes (in comparison to open and laparoscopic surgery), it is widely popular and robotic centers are growing in numbers [51].

Another reason for slow adoption of TE products into clinical practice can be the extensive culture time required for TE constructs. On the other hand, reconstructive urethral surgeries are usually performed on an elective basis, so this should not be a crucial problem.

Regenerative medicine and TE involve cell therapies, gene therapy and biomedical engineering techniques. That is why a TE product is difficult to define due to the extent and complexity it encompasses. These products are now regulated in specific legislations [52].

Any medicinal product that can be used in the EU market requires registration, assessment and approval by the Committee for Advanced Therapies at the European Medicines Agency. The process requires substantial financial, laboratory and human resources, and such products must undergo meticulous regulatory evaluation such as safety testing and confirmation of GMP before approval and widespread use [53]. By definition, a TE product is categorized as “an advanced therapy medicinal product (ATMP) that contains or consists of engineered cells or tissues and is presented as having properties for, or is used in or administered to human beings with a view to regenerating, repairing or replacing a human tissue” [54]. Consequently, TE products must undergo strict and complex assessment before entering markets to be widely and commonly used in clinical practice. This can also influence scientists and clinicians considering development and testing of TE urethral replacements, especially when appropriate material such as buccal mucosa or skin is readily available.

## 5. Conclusions

Urethral TE has made slow progress in clinical practice so far. The TE approach is promising and effective in the management of urethral strictures, but in simple cases and short strictures where local tissues or buccal mucosa are available, this should remain the gold standard. Though significant progress to achieving a safe and reliable TE construct has been made, many issues remain that need to be addressed: better design of trials, namely RCTs, better cooperation of research groups and centralization of AMTEP that could lead to reduction of costs and slowly proceed to commercialization of “off the shelf” products. The development and subsequent approval of a TE product require further significant financial and human resources. So far, research of TE of the urethra has not yet been translated into a clinically available material. In the future, 3D bioprinting could help streamline the creation of seeded tubular urethral constructs with the added benefit of patient-tailored designs, increasing the efficiency of generation of these TE urethras for clinical applications.

## Figures and Tables

**Table 1:** Overview of clinical studies.

Material	Technique	Location	Follow-Up in Months (Mean)	Number of Patients	Results	Ref
collagen-based inert matrix - bladder submucosal graft	dorsal onlay	hypospadias - meatus penoscrotal 3 patients - meatus scrotal 1 patient	22	4	1 patient with subglandular fistula repaired using standard techniques all 4 patients as success	[17]
SIS	dorsal onlay	complete urethral stricture	16	1	100% success	[18]
bladder submucosa collagen based inert matrix	ventral onlay	N/A	37	28	24 patients (86%) success 4 patients slight caliber decrease	[19]
Unseeded SIS	endoscopic urethroplasty	bulbar urethral strictures	24	9	2 patients success (25%) 6 patients as failure 1 lost during follow up	[20]
acellular dermis (AlloDerm) + buccal mucosa	dorsal onlay + buccal mucosa ventral cover	4 cm segment of ventral penile urethra	6	1	100% success	[21]
SIS	dorsal onlay technique	bulbar urethras	18	9	8 patients success (89%)	[22]
SIS	dorsal onlay substitution urethroplasty	2 bulbar stricture 3 penile-bulbar stricture	14	5	1 patient success (20%)	[23]
SIS	onlay urethroplasty	10 patients bulbar urethra 31 patients bulbopenile area 9 patients distal penile urethra	31.2	50	40 patients success (80%)	[24]
SIS	14 patients dorsal inlay, 1 patient ventral onlay 5 patients dorsal onlay plus ventral onlay.	Anterior urethral stricture	21	20	17 cases success (85%)	[25]
in vitro cultured urothelial cells on acellular dermis	onlay	scrotal or perineal hypospadias	52	6	6 cases as success (100%)	[26]
autologous tissue-engineered buccal mucosa	dorsal onlay technique	urethral stricture secondary to to lichen sclerosus	33.6	5	0	[27]
acellular bladder matrix (BAMG) and buccal mucosa	ventral onlay	11 patients bulbar stricture 7 pendulous stricture 12 combined	25	30	2 patients lost during follow-up buccal mucosa 15 (100%) BAMG 10 patients success (66%)	[28]
SIS	SIS endoscopically placed	bulbar urethral stricture	14.25	10	8 patients as success (80%)	[29]
seeded tubularised polyglycolic acid: poly(lactide-co-glycolide acid) scaffolds	urethral tubularised posterior urethroplasty	3 patients posterior urethral disruption 2 patients with previous failed posterior urethral repairs	71	5	100% success	[30]
seeded acellular dermis	ventral onlay	scrotal or perineal hypospadias	87	6	100% success	[31]
SIS	dorsal/ventral or dorsal plus ventral onlay	bulbar strictures (non-obliterative)	71	25	19 (76%) success	[32]
SIS	Augmentation urethroplasty Onlay and inlay technique	8 patients bulbar urethra 9 patients bulbopenile area 10 patients distal penile urethra 1 patient after failed hypospadias repair	24.8	28	24 patients success (85%)	[33]
TE autologous oral mucosa graft MukoCell^®^	ventral onlay, dorsal onlay, dorsal inlay and combined	penile in 3 (7.9%) cases, bulbar in 29 (76.3%), peno-bulbar in 6 (15.8%)	55	38	32 patients (84.2%) as success	[34]
TE autologous oral mucosa graft MukoCell^®^	ventral onlay	any etiology, location, length and severity	24	99	success rate 70.8% (46 of 65) and 76.9% (30 of 39)	[35]
acellular TE bovine pericardial patch	dorsal onlay technique	long segment anterior urethral strictures (involving penile and/or bulbar urethra	8	9	8 (88.9%) success	[36]

## Data Availability

Not applicable.
